# The Role of Sense of Coherence During the COVID-19 Crisis: Does it Exercise a Moderating or a Mediating Effect on University Students’ Wellbeing?

**DOI:** 10.1177/21582440231160123

**Published:** 2023-03-15

**Authors:** Vanessa Kulcar, Alexander Kreh, Barbara Juen, Heidi Siller

**Affiliations:** 1 University of Innsbruck, Austria; 2 Medical University of Innsbruck, Austria

**Keywords:** sense of coherence, salutogenesis, pandemic, young adults, mental health

## Abstract

The COVID-19 crisis caused extensive mental health strains. Sense of coherence (SOC) is considered a protective factor for mental health in crisis that might also be decisive during the COVID-19 pandemic, but the mechanisms are not yet well understood. Using longitudinal survey data of 117 Austrian university students collected in 2020, we tested both moderating and mediating effects of SOC for the association of different stressors with later wellbeing. SOC did not buffer but mediated the effects of stressors on wellbeing. Students especially suffered from reduced feelings of manageability when confronted with financial strains, dissatisfying study situations, or disrupted plans. Supporting them in managing the difficulties of the crisis should therefore be considered a crucial part of psychosocial support.

## Introduction

The COVID-19 pandemic that spread across the world in early 2020 is associated with intense mental health strains (Salari et al., 2020; Xiong et al., 2020). Young people like university students were psychologically burdened even before the pandemic (Patel et al., 2007; Twenge et al., 2019; Xiao et al., 2017) and were especially vulnerable to the impact of the COVID-19 crisis on their mental health (Liu et al., 2020; Xiong et al., 2020). Hence, it is crucial to investigate protective factors to support this group in maintaining their mental wellbeing during the crisis.

A theory that focuses on health promotion and health maintenance is salutogenesis (Antonovsky, 1979, 1993). Antonovsky proposed that health should be understood on a multi-dimensional continuum in place of a dichotomy of health and illness. He emphasized the importance of comprehending individuals with respect to their life stories, including the individual’s strengths and coping strategies as well as considering individual adaptation to the environment (Antonovsky, 1997). In this sense, salutogenesis also illustrates that individual experiences affect how we manage and use resources and how we cope with stressors (Bauer et al., 2020). Within the framework of salutogenesis, the concept of the sense of coherence (SOC) was developed (Antonovsky, 1979; Blättner, 2007; Lindström & Eriksson, 2005). SOC represents a general and individual global orientation that is dynamic on the one hand but expected to reach stability in adulthood on the other hand (Antonovsky, 1997; Mc Gee et al., 2018a). SOC illustrates how individuals select and employ adaptive strategies in stressful situations (Antonovsky, 1997; Blättner, 2007). It includes three facets: First, the behavioral component, manageability, attenuates possibilities and resources to counteract stressors. Second, the cognitive facet, comprehensibility, relates to cognitively understanding internal or external stimuli. This facet structures and organizes the “chaos” surrounding stressors. The third facet is the aspect of meaningfulness which represents the motivational component of SOC (Antonovsky, 1987; Eriksson & Mittelmark, 2017). Experiencing consistency strengthens comprehensibility, whereas involvement in meaningful and socially sensible decision-making processes enhances meaningfulness. Manageability increases when individuals experience a balance between demands (Bauer et al., 2020).

### SOC During the COVID-19 Crisis

SOC is repeatedly studied as a potentially protective factor during times of crisis. The COVID-19 pandemic poses no exception. While a positive association with mental health, especially during stressful and traumatic experiences, has been repeatedly shown before (Eriksson & Lindström, 2006; Schäfer et al., 2019) and during the pandemic (Généreux et al., 2020; Mana, Bauer, et al., 2021; Mana, Super, et al., 2021; Schäfer et al., 2020), the underlying mechanisms are not yet fully uncovered. Particularly, differentiation between moderating and mediating effects of SOC is important as the mechanisms have different implications for the relation between SOC and mental health. A mediating effect suggests that adversities affect SOC which decreases as a result. When people experience weaker SOC, their mental wellbeing also decreases. In this case, mental health impacts are similar for people with higher and lower SOC and wellbeing fluctuates simultaneously with SOC. However, people with higher initial SOC might experience fewer mental health impairments during a crisis as it takes longer for SOC to be depleted. Contrarily, a moderating effect indicates that mental health effects of events are different depending on initial SOC levels. If SOC is assumed to be protective for mental health, a buffering effect is to be expected. Specifically, negative effects of stressors are expected to be larger for people with lower levels of SOC compared to people with higher levels of SOC. Put differently, people with stronger SOC should suffer less from the same negative event.

During the COVID-19 crisis, some studies indicated a moderating effect. In a study on 2,784 adults (65.4% women) during the third week of the initial lockdown in Italy in 2020, Barni et al. (2020) detected negative effects of knowing infected persons on wellbeing in people with weaker SOC, supporting a buffering effect. They additionally observed the reverse moderating effect of SOC for the association of fear of infection with wellbeing: People with stronger SOC reported lower wellbeing when they feared a COVID-19 infection but the effect was less pronounced in people with weaker SOC. In a preprint, Hirano et al. (2020) surveyed 570 university students (59% women) and reported a moderating effect of SOC in male Japanese students, but not in female students. Male students with anxiety of COVID-19 reported fewer mental health impairments when their SOC score was higher, supporting a buffering effect in men only. In their study on SOC and psychopathological symptoms in a sample of 1,591 German-speaking participants, Schäfer et al. (2020) showed that higher levels of pre-pandemic SOC were associated with fewer changes in psychopathological symptoms during the pandemic. However, SOC was not stable and decreased in people with high levels of pre-pandemic stress. Other studies examined SOC as a potential mediator for the associations between pandemic-related stressors and mental health. For example, in their study on 907 adults (58% women) in Poland, Dymecka et al. (2022) detected a mediating effect of SOC for the association between stress and life satisfaction during the COVID-19 crisis. Similarly, in a study on 441 health care providers (71% women) in Palestine, Veronese et al. (2022) found that SOC mediated the effects of COVID stress on symptoms of trauma and burnout.

Overall, studies indicating moderating effects of SOC during the COVID-19 pandemic often had small effect sizes and findings were inconsistent with effects differing for genders (Hirano et al., 2020) or contrasting directions of the interaction (Barni et al., 2020). Therefore, based on the available studies, a buffering effect of SOC for mental health impacts of the COVID-19 crisis cannot be verified. For mediation effects, results are less diverse but studies comparing moderating and mediating effects are scarce; particularly as even before the pandemic only few studies examined moderator and mediator effects simultaneously and partly resulted in inconclusive outcomes. That is, Veronese et al. (2013) confirmed both a moderating and mediating effect of SOC for the association of traumatic stress and psychological wellbeing in war contexts as did Hansen et al. (2018) regarding occupational stress and sleep problems. Contrarily, Mc Gee et al. (2018b) only obtained partial support for both mechanisms in their study on chronic stress and early life adversity in Swiss older adults. SOC did not buffer the effects of early life adversity on psychological health and wellbeing and only one subscale was a significant buffer for the effects of chronic stress. Meanwhile, SOC mediated the effects of chronic stress and early life neglect but not of early life abuse on mental health. It can thus be concluded that the role of SOC for mental health needs clarification. The COVID-19 pandemic further emphasizes the demand for research in this area due to its mental health strains.

### The Present Study

To increase the understanding of the mechanisms through which SOC affects mental health during the COVID-19 pandemic, we explored the two potential effects of SOC in one sample and on a longitudinal timescale: First, we assumed a protective effect of SOC regarding mental health strains in stressful situations, which would be shown by a moderating effect of SOC. In this sense, we hypothesized that the negative effects of stressors on wellbeing are amplified in people with weaker SOC compared to people with stronger SOC. This effect would imply that SOC protects against mental health impairment. Second, we assumed that SOC has a mediating effect. In this case, people exposed to stressors would report decreased SOC, which is in turn associated with decreased psychological wellbeing. In contrast to the moderating effect, a mediating effect does not imply any protection from mental health strains induced by stressors. If SOC mediates the effects on mental health, it can be considered one aspect or a precursor of mental health but not a protective factor.

**Figure 1.**
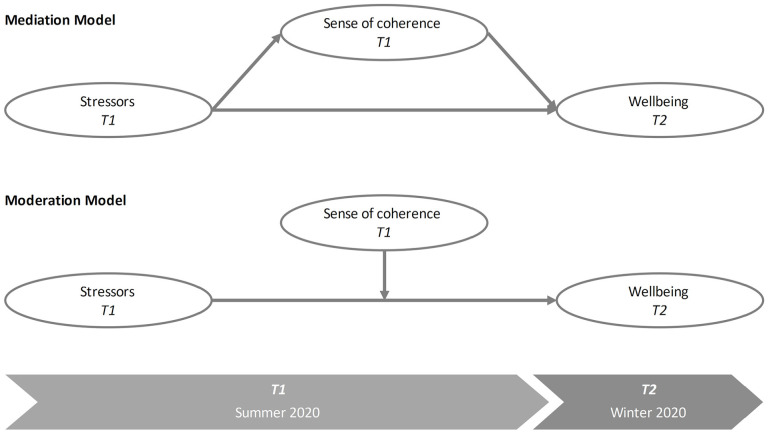
Research plan.

We used longitudinal data from university students to analyze moderating as well as mediating effects of SOC on wellbeing. We collected data in summer 2020 (T1), at a time of low rates of COVID-19 infections, and again in autumn 2020 (T2) when the number of infections increased and the second wave of COVID-19 began. We utilized different stressors and SOC reported at T1 to predict psychological wellbeing approximately 4 months later (T2), as presented in Figure 1.

## Method

The data used in this article are part of a longitudinal study on the psychological effects of the COVID-19 crisis on university students. Participants from two universities in Innsbruck, Austria, were recruited for this study. The study was designed as an online survey. It was conducted in accordance with the Declaration of Helsinki and approved by the Review Board for Ethical Questions of the University of Innsbruck. The ethics committee of the Medical University of Innsbruck declared the study exempt from ethical review. All participants provided informed consent before participation.

### Data Collection

Between July 8th and August 29th, 2020 (T1), 480 students completed the survey. During this first survey period, there were on average twelve new infections per 100,000 people in 7 days in Austria (Bundesministerium für Soziales, Gesundheit, Pflege und Konsumentenschutz, 2022). The first wave of the pandemic had passed during this time and pandemic measures and restrictions were largely loosened. Then, the case rates increased until the next data collection between November 23rd and December 21st, 2020 (T2), with an average of 269 new infections per 100,000 people and 7 days. Associated with this increase in case rates, rigorous measures were reintroduced to contain the spread of the pandemic during the second data collection. Of the participants at T1, 117 also participated at T2 which allowed longitudinal analysis. To match participants at both data collection points, students entered an anonymized code, which was either chosen by themselves or based on an instruction for selecting a combination of characters to ensure anonymity. Ninety-seven participants were female (82.9%), 19 were male (16.2%), and 1 person did not identify with binary genders (0.9%). The average age was 23.97 years (*SD* = 5.35). Students were mostly living in Austria during the survey period (*n* = 81, 69.2%), followed by Germany (*n* = 25, 21.4%), Italy (*n* = 9, 7.7%), and other countries (*n* = 2, 1.7%). Most students were living with their parents (*n* = 46, 39.3%) or in shared housing (*n* = 37, 31.6%). Fewer students were living alone (*n* = 16, 13.7%) or with their partner (*n* = 18, 15.4%). For a description of the full sample at T1, see Supplemental Appendix.

### Instruments

Students provided information on socio-demographic data such as age, gender, and country of residence at the beginning of the surveys.

*Wellbeing* was measured using a German translation of the WHO-5 (Bech, 1999; Brähler et al., 2007). This instrument consists of five items, which were rated on a six-point Likert response format (0—*never* to 5—*all the time*). The scale was assessed by the sum of all items, with higher scores indicating higher levels of wellbeing. Cronbach’s α = .84 indicated good internal consistency of the scale in our sample.

*Sense of coherence* was measured with the German 13 item scale SOC-13 (Singer & Brähler, 2007). The items were rated on a seven-point Likert response format with varying verbal anchoring. For this study, we used the global scale (α = .83) and the three subscales comprehensibility (five items, α = .66), manageability (four items, α = .68), and meaningfulness (four items, α = .65) by computing sum scores for each scale. Higher scores indicate higher levels of SOC.

*Stressors* during the COVID-19 crisis were assessed using four single items. Based on a pre-study and other results during the pandemic (American Psychological Association, 2020; Cao et al., 2020; Salari et al., 2020; Shanahan et al., 2020; Son et al., 2020; Xiong et al., 2020), four stressors were chosen: knowing infected people, experiencing financial strains, being dissatisfied with the study situation, and experiencing disruptions of plans. This allowed a comparison of the effects of SOC for various kinds of stressors and thereby testing the consistency of effects. All stressors were assessed dichotomously in the analysis (coded as 0—*stressor absent* and 1—*stressor present*). *Knowing infected people* was measured by asking if participants personally knew someone who tested positive for COVID-19. Respondents could agree or disagree with the statement. *Financial strains* were measured by asking participants if their financial situation was worse compared to before the pandemic. The item was answered on a five-point Likert response format (1—*does not apply at all* to 5—*does apply completely*). For the purpose of this study, answers one to three were considered a rejection and answers four or five were considered an approval. To assess the *study situation*, students were asked if they were satisfied with the current execution of university courses that were largely shifted to online formats. The answering format consisted of the same five-point Likert response format as for the item on financial strains. For dichotomization of this item, answers one to two were considered a rejection, answers three to five were considered an approval. *Disruption of plans* was measured by asking if the effects of the COVID-19 crisis disrupted important plans for the future. The answer format included short-, middle-, and long-term plans, which were summarized for the analysis of this study.

### Data Analysis

We used regression analyses to study the effects of SOC or stressors at T1 on wellbeing at T2 separately. We then conducted moderation and mediation analyses using PROCESS by Hayes (2018). In simple moderation analyses, moderating effects of SOC on the effects of stressors on wellbeing were assessed. Since no cut-off values for different levels of SOC exist, significant interaction effects were examined using the pick-a-point approach at the 16th, 50th, and 84th percentile (Hayes, 2018). Mediation analyses were conducted as simple and as parallel mediations. Indirect effects were estimated using 95% confidence intervals of 10,000 bootstrap samples. Using G*Power (Faul et al., 2009), post-hoc power analysis for the moderation analyses was conducted. Assuming an additional explanation of 10% of variance by the moderator and α = .05, a power of (1 − β) = .95 was achieved based on the longitudinal sample (*N* = 117). For mediation analysis, the application by Schoemann et al. (2017) was employed for post-hoc power analysis. The longitudinal sample had a limited power of (1 − β) = .59 to detect a simple mediating effect assuming small effects of the stressor on SOC and wellbeing and a large effect of SOC on wellbeing. We examined the effects for each stressor separately and did not include the other stressors as covariates because of further reduced power when using multiple predictors. However, we repeated all analyses utilizing the larger cross-sectional sample of T1 (*N* = 480) to verify our results. This allowed the power of (1 − β) = .99 for detecting a moderating effect that increases explained variance by 5% and the power of (1 − β) = .99 to detect a simple mediating effect. Further, we included all predictors as covariates in the cross-sectional analyses. The results of these cross-sectional analyses are presented in detail in the Supplemental Appendix.

## Results

During T1 in summer 2020, 57 students (48.7%) knew someone who tested positive for COVID-19. Twenty-four students (20.5%) experienced financial strains due to COVID-19 and 47 students (40.2%) reported being dissatisfied with the study situation. A majority of participants (*n* = 85, 72.6%) reported disruptions of plans. At T1, mean SOC was *M* = 58.64 (*SD* = 12.40) for the global scale, *M* = 20.81 (*SD* = 5.65) for comprehensibility, *M* = 17.96 (*SD* = 4.92) for manageability, and *M* = 19.87 (*SD* = 4.12) for meaningfulness. Mean wellbeing, as measured with the WHO-5, was *M* = 12.13 (*SD* = 5.11) during T2 in winter 2020.

Effects of stressors associated with the COVID-19 pandemic on wellbeing 4 months later were first explored using multiple linear regression analysis. The stressors explained 23% of variance (*F*(4, 112) = 8.27, *p* < .001, *R*^2^ = .23). Financial strains (β = −.33, *p* < .001) and being dissatisfied with the study situation (β = −.19, *p* = .039) were associated with decreased wellbeing. There were no effects of knowing infected people and disrupted plans on wellbeing (see Table 1).

**Table 1.** Regression Analysis for the Effects of Stressors on Later Wellbeing.

	*B*	*SE*	β	*p*-Value
Constant	14.73	0.95		<.001
Infected people	0.43	0.86	0.04	.624
Financial strains	−4.17	1.10	−0.33	<.001
Study situation	−1.92	0.92	−0.19	.039
Disrupted plans	−1.62	1.04	−0.14	.123

*Note. N* = 117; stressors: 0 = stressor absent, 1 = stressor present.

Sense of coherence predicted 30% of the variance of wellbeing (*F*(1, 115) = 48.32, *p* < .001, *R*^2^ = .30) while having a strong positive effect (*B* = 0.22, *SE* = 0.03, β = .54, *p* < .001). When the different facets of SOC were examined as individual predictors (*F*(3,113) = 16.86, *p* < .001, *R*^2^ = .31), there were significant effects of meaningfulness (*B* = 0.28, *SE* = 0.11, β = .23, *p* = .015) and manageability (*B* = 0.38, *SE* = 0.12, β = .36, *p* = .002) but not of comprehensibility (*B* = 0.07, *SE* = 0.11, β = .08, *p* = .540).

### SOC as Moderator

**Figure 2.**
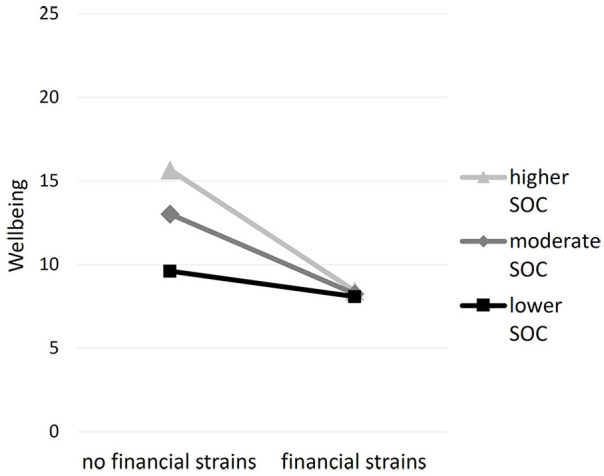
Wellbeing in students with and without financial problems and with lower, moderate, and higher levels of SOC.

To assess our first assumption—SOC as a protective factor for mental health—we conducted moderation analyses. We used stressors as predictors, SOC as moderator, and wellbeing 4 months later as outcome. There were no significant interaction effects for knowing infected people (*B* = 0.09, *SE* = 0.07, *F*(1, 113) = 1.97, Δ*R*^2^ = .01, *p* = .163), dissatisfaction with the study situation (*B* = −0.05, *SE* = 0.07, *F*(1, 113) = 0.56, Δ*R*^2^ = .00, *p* = .455), and disrupted plans (*B* = −0.09, *SD* = 0.07, *F*(1, 113) = 1.36, Δ*R*^2^ = .01, *p* = .246) on wellbeing. There was a significant interaction effect for financial strains (*B* = −0.23, *SD* = 0.08, *F*(1, 113) = 8.86, Δ*R*^2^ = .05 *p* = .004). The financial situation did not have an effect on wellbeing for students with lower levels of SOC (SOC_16_ = 45.76, *B* = −1.52, *SE* = 1.12, *p* = .178), but students with moderate (SOC_50_ = 60.00, *B* = −4.76, *SE* = 1.08, *p* < .001) and higher (SOC_84_ = 71.00, *B* = −7.27, *SE* = 1.64, *p* < .001) levels of SOC reported decreased wellbeing (see Figure 2). When the analyses were repeated using the larger cross-sectional sample, no significant moderating effects of SOC were found (see Supplemental Appendix). The hypothesis that SOC buffers the effects of stressors on wellbeing was thus rejected.

### SOC as Mediator

**Figure 3.**
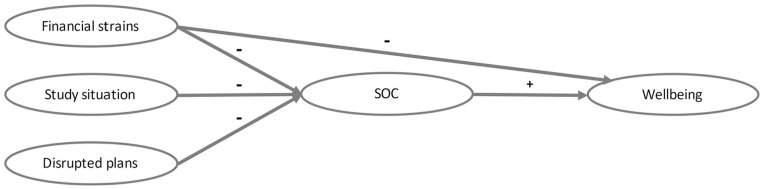
Significant direct and indirect effects of stressors on wellbeing mediated by SOC. *Note*. Knowing infected people did not have significant effects and was therefore not included in the figure.

For our second assumption—SOC as one aspect or a precursor of mental health—we tested if SOC mediated effects on wellbeing. Stressors were used as predictors and wellbeing 4 months later as outcome. Knowing infected people was associated with increased levels of SOC (*B* = 4.57, *SE* = 2.26, *p* = .046) and higher levels of SOC were associated with increased wellbeing (*B* = 0.22, *SE* = 0.03, *p* < .001), resulting in an indirect effect (*B* = 1.02, *SE* = 0.51, 95% CI [0.03, 2.05]). However, the effect of knowing infected people on SOC did not meet the significance threshold of *p* < .0125 after correcting for multiple testing. There was no direct effect of knowing infected persons on wellbeing (*B* = 0.21, *SE* = 0.81, *p* = .801, 95% CI [−1.41, 1.82]). In the cross-sectional sample, there was neither a direct nor an indirect effect of knowing infected persons. Financial strains had a negative direct effect on wellbeing at T2 (*B* = −3.28, *SE* = 0.99, *p* = .001, 95% CI [−5.24, −1.32]) and a negative indirect effect mediated by SOC (*B* = −1.70, *SE* = 0.63, 95% CI [−3.10, −0.62]). Thereby, financial strains were associated with decreased levels of SOC (*B* = −8.83, *SE* = 2.73, *p* = .002), which in turn was associated with wellbeing (*B* = 0.19, *SE* = 0.03, *p* < .001). An equivalent indirect effect was detectable for dissatisfaction with the study situation. Dissatisfaction was associated with decreased SOC (*B* = −6.01, *SE* = 2.28, *p* = .010), which was associated with later wellbeing (*B* = 0.21, *SE* = 0.03, *p* < .001). The indirect effect was significant (*B* = −1.26, *SE* = 0.48, 95% CI [−2.26, −0.34]). There was no direct effect of dissatisfaction with the study situation on wellbeing (*B* = −1.51, *SE* = 0.83, *p* = .070, 95% CI [−3.16, 0.13]). Disrupted plans equally did not have a direct effect on wellbeing (*B* = −1.54, *SE* = 0.94, *p* = .103, 95% CI [−3.40, 0.32]) but were associated with decreased SOC (*B* = −9.01, *SE* = 2.44, *p* < .001), which was associated with wellbeing (*B* = 0.21, *SE* = 0.03, *p* < .001). The indirect effect was significant (*B* = −1.86, *SE* = 0.55, 95% CI [−2.96, −0.81]). The effects are illustrated in Figure 3. In analyses of the cross-sectional data, there was no direct effect of the stressors financial strains, dissatisfaction with the study situation, and disruption of plans on wellbeing at the same time. Instead, all stressors but knowing infected people had indirect negative effects via decreased levels of SOC.

### The Three Facets of SOC

We differentiated between the three facets of SOC (manageability, comprehensibility, and meaningfulness) and repeated the analyses. Thereby we tested effects that yielded significant results for the global SOC separately for all three facets. First, we repeated the moderation analysis with financial problems as predictor, wellbeing as outcome, and all three components of SOC as moderators. The analyses were conducted separately for each moderator. There was no significant moderating effect of meaningfulness (*B* = −0.35, *SE* = 0.25, *F*(1, 113) = 2.03, Δ*R*^2^ = .01, *p* = .157) but manageability had a significant moderating effect (*B* = −0.52, *SE* = 0.19, *F*(1, 113) = 7.77, Δ*R*^2^ = .04, *p* = .006) and so did comprehensibility (*B* = −0.52, *SE* = 0.17, *F*(1, 113) = 9.79, Δ*R*^2^ = .06, *p* = .002). In both cases, there was no effect of financial strains on wellbeing for students with lower levels of SOC (manageability_16_ = 12.88, *B* = −1.82, *SE* = 1.15, *p* = .118; comprehensibility_16_ = 14.00, *B* = −1.25, *SE* = 1.29, *p* = .333). Students with moderate (manageability_50_ = 18.00, *B* = −4.49, *SE* = 1.03, *p* < .001; comprehensibility_50_ = 21.00, *B* = −4.92, *SE* = 1.03, *p* < .001) and higher (manageability_84_ = 23.00, *B* = −7.10, *SE* = 1.61, *p* < .001; comprehensibility_84_ = 26.00, *B* = −7.54, *SE* = 1.52, *p* < .001) levels of manageability and comprehensibility reported decreased wellbeing when experiencing financial strains.

Next, we conducted parallel mediation analyses with all facets of SOC as mediators, the stressors financial strains, study situation, and disrupted plans as predictors, and wellbeing as outcome. Even though there was no effect of comprehensibility on wellbeing in the regression analysis, all three facets were included to account for potential confounding effects. Again, there was a negative direct effect of financial strains on wellbeing at T2 (*B* = −3.16, *SE* = 0.99, *p* = .002, 95% CI [−5.14, −1.18]). Additionally, there was an indirect effect through manageability (*B* = −1.03, *SE* = 0.51, 95% CI [−2.19, −0.20]). Financial strains were associated with reduced levels of manageability (*B* = −3.25, *SE* = 1.09, *p* = .004), which in turn were associated with wellbeing (*B* = 0.32, *SE* = 0.12, *p* = .007). We found no indirect effects via comprehensibility (*B* = −0.23, *SE* = 0.37, 95% CI [−1.05, 0.48]) and meaningfulness (*B* = −0.56, *SE* = 0.39, 95% CI [−1.48, 0.02]). Further, there were no direct effects of dissatisfaction with the study situation (*B* = −1.62, *SE* = 0.84, *p* = .055, 95% CI [−3.28, 0.04]) and disrupted plans (*B* = −1.61, *SE* = 0.94, *p* = .089, 95% CI [−3.47, 0.26]) on wellbeing. For both stressors, there were significant indirect effects mediated by manageability (study situation *B* = −0.88, *SE* = 0.40, 95% CI [−1.78, −0.20]; disrupted plans *B* = −1.16, *SE* = 0.47, 95% CI [−2.20, −0.35]) but not by comprehensibility (study situation *B* = −0.11, *SE* = 0.34, 95% CI [−0.88, 0.51]; disrupted plans *B* = −0.16, *SE* = 0.45, 95% CI [−1.09, 0.75]) or by meaningfulness (study situation *B* = −0.16, *SE* = 0.25, 95% CI [−0.68, 0.33]; disrupted plans *B* = −0.47, *SE* = 0.33, 95% CI [−1.22, 0.06]). Dissatisfaction with the study situation was associated with decreased levels of manageability (*B* = −2.49, *SE* = 0.90, *p* = .007) which was associated with wellbeing (*B* = 0.35, *SE* = 0.12, *p* = .003). Likewise, disrupted plans were associated with decreased levels of manageability (*B* = −3.24, *SE* = 0.98, *p* = .001), which were associated with decreased wellbeing (*B* = 0.36, *SE* = 0.12, *p* = .003). In the cross-sectional data, the indirect effects of stressors were mediated by all factors of SOC. The only exception was meaningfulness, which did not mediate the effect of dissatisfaction with the study situation on wellbeing.

### Effects of Gender and Age

We explored gender and age effects on associations between SOC and wellbeing. The analyses exploring gender effects excluded one person who did not identify as male or female due to the lacking sample size for non-binary students. Binary gender did not moderate the effects of the stressors on SOC (knowing infected people *B* = −4.75, *SE* = 6.36, *F*(1, 112) = 0.56, Δ*R*^2^ = .00, *p* = .457, financial problems *B* = 11.03, *SE* = 8.07, *F*(1, 112) = 1.87, Δ*R*^2^ = .02, *p* = .175; dissatisfaction with the study situation *B* = 4.00, *SE* = 6.81, *F*(1, 112) = 0.34, Δ*R*^2^ = .00, *p* = .559; disruption of plans *B* = 9.02, *SE* = 6.12, *F*(1, 112) = 2.18, Δ*R*^2^ = .02, *p* = .143), neither did age (knowing infected people *B* = 0.39, *SE* = 0.44, *F*(1, 113) = 0.81, Δ*R*^2^ = .01, *p* = .371; financial problems *B* = −0.68, *SE* = 0.83, *F*(1, 113) = 0.68, Δ*R*^2^ = .01, *p* = .412; dissatisfaction with the study situation *B* = 0.08, *SE* = 0.46, *F*(1, 113) = 0.03, Δ*R*^2^ = .00, *p* = .868; disruption of plans *B* = 0.08, *SE* = 0.45, *F*(1, 113) = 0.03, Δ*R*^2^ = .00, *p* = .866). Further, there were no interaction effects of gender (*B* = 0.07, *SE* = 0.10, *F*(1, 112) = 0.54, Δ*R*^2^ = .00, *p* = .465) or of age (*B* = −0.01, *SE* = 0.01, *F*(1, 113) = 0.38, Δ*R*^2^ = .00, *p* = .538) for the association of SOC and wellbeing.

## Discussion

This study investigated two potential mechanisms for the effects of SOC on mental health in a sample of university students in Austria. We analyzed SOC as a mediator and as a moderator variable for different COVID-19 associated stressors in longitudinal data and found support for SOC as a mediator but not a buffer for mental health effects.

### The Role of SOC for Mental Health

SOC is seen as a global orientation of individuals (Antonovsky, 1987) and intended to provide a theoretical basis for health promotion. SOC offers a unique perspective on health by including cognitive, motivational, and behavioral aspects that contribute to wellbeing and health (García-Moya & Morgan, 2017). However, there are some difficulties when it comes to the concept of SOC, such as overlaps with other concepts and its undetermined status as a moderator or a mediator variable in mental health. The overlaps with similar concepts—such as resilience—have already been discussed (Grevenstein et al., 2016) and were not within the scope of this study. Studies on the second challenge, the status as moderator or mediator variable, have resulted in often conflicting outcomes. Nevertheless, it is substantial to determine the mechanisms of SOC in mental health to attune interventions, and this study was designed to contribute to this unsettled question.

We obtained no support for a buffering effect of SOC for mental health impacts, but SOC mediated the effects of stressors on wellbeing. We, therefore, argue that SOC can be considered a part of mental health based on a broader definition than merely feeling well. A broader definition of mental health includes a high level of psychological functioning and the ability to manage stressful situations (Maercker & Zoellner, 2004) and SOC fundamentally contributes to both as it enables the individual to successfully handle small disruptions. SOC is assumed to be relatively stable during adulthood (Antonovsky, 1997) but major life challenges can impact SOC and therefore other aspects of wellbeing. Our results indicate that the COVID-19 crisis constituted such a major event for some students. Their SOC was disrupted by different pandemic stressors which was straining their mental health.

So far, it is unknown if these disruptions are short-termed or if there will be persistent effects on SOC and mental health. The crisis was still ongoing during the second data collection and the effects in this study can therefore be classified as short-term reactions. It is possible that levels of SOC and wellbeing recover over time and might even increase if the crisis is mastered successfully. Supporting the assumption of potential recovery, Bonanno et al. (2008) reported that about 20% of severe acute respiratory syndrome (SARS) survivors who experienced reduced levels of psychological functioning shortly after their disease recovered within 18 months. Similarly, posttraumatic growth—the experience of positive psychological change after trauma—is also known to emerge as a long-term effect after a crisis (Zoellner & Maercker, 2006). This underlines that long-term studies are essential to fully assess the effects of SOC during and after crises.

### Disrupted Expectations of Coherence During COVID-19

While we did not observe the hypothesized buffering effect of SOC, there was a moderating effect in the opposite direction. Students with moderate to higher levels of SOC experienced more substantial negative effects on their wellbeing when confronted with financial strains during the COVID-19 crisis. Students with lower levels of SOC experienced no effect of financial strains on mental health. This finding is in coherence with SOC as a concept that considers life experiences. Thus, we assume that students with lower SOC already have lower wellbeing independent of acute stressors. They did not make the experience that situations are manageable and comprehensible before the pandemic, so they did not have expectations to be disrupted by the crisis. This non-significant effect of financial strains on wellbeing in students with lower levels of SOC should not be equated with a prompt to neglect the needs of those students. On the contrary, this group of students needs ongoing support to foster SOC, related to the effects of the pandemic and beyond that. While expectations to manage stressors could not be disappointed in students with weaker SOC, students with stronger SOC were accustomed to manageable and comprehensible stressors prior to the pandemic. During the pandemic, the financial strains of students with stronger SOC might have interfered with these expectations of coherence. They might have interpreted financial strains as a threat to satisfying basic needs and as a profound disruption of their abilities regarding manageability and comprehensibility. One indicator supporting this assumption is the number of students who chose to move back to their parents after the first lockdown due to financial strains (Yorguner et al., 2021). Such a decision might be associated with a loss of independence which might impact wellbeing, especially for those with previously higher levels of SOC who were used to being able to manage challenges successfully.

Taken together, our results highlight the importance of supporting university students during the COVID-19 pandemic by strengthening their SOC. Especially financial security and predictability regarding their general and educational situation seem to be important aspects for students to maintain higher SOC and wellbeing. Regarding the three facets of SOC, experiencing the situation as manageable appeared to be decisive for students during this pandemic. Accordingly, interventions to support university students during the COVID-19 pandemic should focus on providing them the opportunity to actively address, modify, and improve their situation themselves, especially regarding their study situation and plans for the future. One pathway to achieve this is providing them with appropriate resources to cope with pandemic stressors. Previous results indicate that the promotion of social support might be one pathway to increasing the perceived manageability of crises and thereby protecting mental health. For instance, Mana, Bauer, et al. (2021) reported that family support, as well as trust in leaders and institutions, mediated the association of SOC with emotional, social, and psychological wellbeing during the COVID-19 crisis. Also, during the previous health crisis of the SARS pandemic, Bonanno et al. (2008) determined social support as a predictor of SARS survivors’ psychological resilience and recovery. Other studies indicated the benefits of psychoeducational (Hood et al., 2021) and life skill courses (Maddah et al., 2021) for university students during the COVID-19 pandemic that might also strengthen SOC, especially manageability. However, our results also imply that psychosocial interventions should not only focus on specific crises but need to support SOC independent of acute stressors, particularly considering students with weak SOC.

### Impact of Circumstances

Not all stressors included in this study had negative effects on SOC and wellbeing. Contrarily to other studies, for example in Italy (Barni et al., 2020), there was no negative effect of knowing infected people on wellbeing and SOC. This might be related to the health system in Austria. Compared to some other countries (Buzelli & Boyce, 2021), the Austrian health system was able to avoid shortages of hospital beds, also due to lessons learnt from neighboring countries. This sense of relative safety in terms of the national health system might have prevented negative effects in addition to the proclamation that younger people typically do not experience a severe course of disease after infection (Mueller et al., 2020). These variations in findings between different countries again highlight that interventions should not only focus on individuals and their psychological responses to crises but that the circumstances are equally important.

Further, others have shown that the effects were dependent on gender, that is, Hirano et al. (2020) detected a moderating effect of SOC only in male students. We did not find that gender or age changed the effects of SOC in our study. While in our study male participants reported slightly higher SOC and other studies found higher wellbeing in men (Barni et al., 2020), the overall effect of SOC and wellbeing does not seem to differ depending on gender. Likewise, age did not change the association of SOC and wellbeing. These results indicate that SOC indeed represents a global orientation independent of demographic variables.

### Strength and Limitations

A strength of this study is the longitudinal period. As many studies use cross-sectional samples, this article adds significantly to their results. Further, most studies evaluated either the moderating or the mediating effects of SOC on mental health. Comparing both effects in the same sample provides the opportunity of exploring the relevance of SOC and its role in mental health. Additionally, previous studies rarely differentiated between the three facets of SOC. By analyzing the effects of each component, we were able to identify which was most decisive for university students’ mental health.

Still, this study is not without limitations. The sample is a convenience sample of university students. Therefore, results cannot be generalized to other population groups. Additionally, the sample size of *N* = 117 offers only limited power for detecting small effects. The small sample size is owed to the small number of participants who took part in the first as well as the second data collection and could be matched by their codes. Results might further be skewed by differences between people taking part in the study repeatedly and those not doing so. We counteracted this by repeating our analyses utilizing the larger cross-sectional sample of T1. Another limitation concerns the measurement of stressors and SOC. Antonovsky’s scale was criticized early on for its overlap with mental health outcomes and for its unstable factorial structure and overlapping factors (Flannery & Flannery, 1990). However, the scale is still used regularly in research and to date did not get widely replaced by a more recent revised scale (Bachem & Maercker, 2018). At last, we did not employ objective measures for stressors. Therefore, it cannot be ruled out that the association between stressors and SOC is partly based on students with lower levels of SOC interpreting their life events more negatively than students with higher SOC.

## Conclusion

As supported by our study, SOC should be considered an indicator of peoples’ current state of mental health and less a protective factor. Experiencing life as coherent did not buffer COVID-19 impacts on mental health; instead, declining SOC in response to pandemic stressors represented a first sign of their mental health impacts. Our results highlight the need to support students in experiencing their lives as manageable, comprehensible, and meaningful during large-scale crises like the COVID-19 pandemic. But not only during crises but also in their everyday lives, increasing students’ SOC is essential to equip them for navigating challenges. Therefore, we argue that, apart from interventions which train individuals on how to manage stressors and how to maintain health, it is crucial to provide conditions that enable young adults to experience manageability, comprehensibility, and meaningfulness.

## Supplemental Material

sj-docx-1-sgo-10.1177_21582440231160123 – Supplemental material for The Role of Sense of Coherence During the COVID-19 Crisis: Does it Exercise a Moderating or a Mediating Effect on University Students’ Wellbeing? Click here for additional data file. Supplemental material, sj-docx-1-sgo-10.1177_21582440231160123 for The Role of Sense of Coherence During the COVID-19 Crisis: Does it Exercise a Moderating or a Mediating Effect on University Students’ Wellbeing? by Vanessa Kulcar, Alexander Kreh, Barbara Juen and Heidi Siller in SAGE Open
