# Supplementary material for: The Role of Sense of Coherence During the COVID-19 Crisis: Does it Exercise a Moderating or a Mediating Effect on University Students’ Wellbeing?
Source: Sage Open. 2023 Mar 15;13(1):21582440231160123. doi: 10.1177/21582440231160123 (PMC10018232; doi:10.1177/21582440231160123)
Supplement: sj-docx-1-sgo-10.1177_21582440231160123 – Supplemental material for The Role of Sense of Coherence During the COVID-19 Crisis: Does it Exercise a Moderating or a Mediating Effect on University Students’ Wellbeing? [file sj-docx-1-sgo-10.1177_21582440231160123.docx]

**The Role of Sense of Coherence during the COVID-19 Crisis:
Does it Exercise a Moderating or a Mediating Effect on University Students’ Wellbeing?**

**Appendix: Cross-sectional analysis**

To test our results in a larger sample, we repeated the moderation and mediation analyses using the cross-sectional sample of students at T1 (*N* = 447). Mean age for this sample was 23.85 years (*SD* = 4.55). Most participants (*n* = 338) were female (75.6%), 105 were male (23.5%), and four participants did not identify with binary genders (0.9%). Three hundred and eight participants were living in Austria at the time of the data collection (68.9%), 85 in Germany (19.0%), and 54 in other countries (12.1%). Most were living with their parents (*n* = 177, 39.6%) or in shared housing (*n* = 136, 30.4%), followed by 85 participants living with their partner (19.0%) and 49 living alone (11.0%).

In the cross-sectional sample, 52.8% knew someone infected with COVID-19, 18.3% experienced financial strains, 41.8% were dissatisfied with the study situation, and 78.7% reported disrupted plans. All stressors were entered as covariates in the analyses. Prevalence of stressors did not differ significantly between students who participated only at T1 and students who participated at T1 and T2.

Mean for wellbeing was *M* = 14.68 (*SD* = 4.95), mean for SOC *M* = 60.09 (*SD* = 11.43). Comparing the sub-sample that participated at T2 in the study with the sub-sample that only participated at T1, wellbeing was lower in the repeated measure subsample (*t*(445) = 2.09, *p* = .037) but there was no significant difference in SOC (*t*(445) = 1.59, *p* = .112). Mean for meaningfulness was *M* = 20.73 (*SD* = 3.68), for comprehensibility *M* = 21.26 (*SD* = 5.39), and for manageability *M* = 18.10 (*SD* = 4.67).

SOC was lower in female students (*M* = 59.26, *SD* = 11.77) than in male students (*M* = 62.51, *SD* = 10.06; *t*(441) = -2.56, *p* = .011) but there was no gender difference in wellbeing (*t*(441) = -1.22, *p* = .223). There was no age effect on SOC (*r* = .09, *p* = .062) or wellbeing (*r* = .04, *p* = .356). Further, there were no effects of country (SOC *F*(3,443) = 0.85, *p* = .466; wellbeing *F*(3.443) = 2.17, *p* = .090) or living situation (SOC *F*(3,443) = 1.87, *p* = .135; wellbeing *F*(3,443) = 1.74, *p* = .158).

**Direct effects of stressors and SOC on wellbeing**

Using multiple linear regression analyses, direct effects of stressors and of SOC on wellbeing were examined.

All stressors had significant effects on wellbeing explaining 9% of variance in total (*F*(4,442) = 10.73, *p* < .001, *R^2^* = .09). Knowing infected people was associated with higher wellbeing (*B* = 0.99, *SE* = 0.45, β = .10, *p* = .030), all other stressors were associated with lower wellbeing (financial strains *B* = -2.24, *SE* = 0.59, β = -.18, *p* < .001; dissatisfaction with the study situation *B* = -1.47, *SE* = 0.47, β = -.15, *p* = .002; disrupted plans *B* = -1.14, *SE* = 0.57, β = -.09, *p* = .046).

There were significant effects of all factors of SOC on wellbeing and together they explained 30% of variance (*F*(3,443) = 62.95, *p* < .001, *R^2^* = .30). Meaningfulness (*B* = 0.33, *SE* = 0.06, β = .25, *p* < .001), comprehensibility (*B* = 0.16, *SE* = 0.05, β = .18, *p* = .003), and manageability (*B* = 0.26, *SE* = 0.06, β = .25, *p* < .001) each exerted a small direct effect of wellbeing. The effect of the total SOC scale on wellbeing was large (*B* = 0.23, *SE* = 0.02, β = .54, *p* < .001, *F*(1,445) = 184.63, *R^2^* = .29).

**Moderating effects**

In the cross-sectional sample, there were no significant moderating effects of SOC for the association of stressors with wellbeing:

- There was no moderating effect for knowing infected persons (*B* = -0.01, *SE* = 0.03, *F*(1, 440) = 0.13, Δ*R^2^* = .00, *p* = .716).
- There was no moderating effect for financial problems (*B* = -0.05, *SE* = 0.04, *F*(1, 440) = 1.35, Δ*R^2^* = .00, *p* = .246).
- There was no moderating effect for dissatisfaction with the study situation (*B* = 0.03, *SE* = 0.04, *F*(1, 440) = 0.79, Δ*R^2^* = .00, *p* = .376).
- There was no moderating effect for disrupted plans (*B* = -0.06, *SE* = 0.04, *F*(1, 440) = 1.74, Δ*R^2^* = .00, *p* = .188).

Effects did not change when age and gender were included as covariates.

**Mediating effects**

There were indirect effects mediated by SOC for the stressors financial problems, dissatisfaction with the study situation, and disruption of plans. There were no indirect effects for knowing infected persons. None of the stressors exerted direct effects on wellbeing:

- There were no direct (*B* = 0.68, *SE* = 0.40, *p* = .086, 95%*CI* [-0.10, 1.46]) or indirect (*B* = 0.30, *SE* = 0.22, 95%*CI* [-0.13, 0.75]) effect of knowing infected persons.
- There were no direct (*B* = -0.87, *SE* = 0.53, *p* = .100, 95%*CI* [-1.91, 0.17]) but indirect (*B* = -1.37, *SE* = 0.33, 95%*CI* [-2.06, -0.74]) effects of financial problems.
  Examining the mediating effects of the different SOC factors in a parallel mediation analysis showed that all factors were significant mediators:
  - Indirect effect via meaningfulness (*B* = -0.38, *SE* = 0.19, 95%*CI* [-0.81, -0.06])
  - Indirect effect via manageability (*B* = -0.62, *SE* = 0.22, 95%*CI* [-1.10, -0.24])
  - Indirect effect via comprehensibility (*B* = -0.39, *SE* = 0.17, 95%*CI* [-0.79, -0.10])
- There were no direct (*B* = -0.61, *SE* = 0.41, *p* = .143, 95%*CI* [-1.42, 0.21]) but indirect (*B* = -0.86, *SE* = 0.24, 95%*CI* [-1.35, -0.41]) effects of dissatisfaction with the study situation.
  Examining the mediating effects of the different SOC factors in parallel mediation analysis results in indirect effects via manageability and comprehensibility but not by meaningfulness:
  - No indirect effect via meaningfulness (*B* = -0.12, *SE* = 0.12, 95%*CI* [-0.37, 0.10])
  - Indirect effect via manageability (*B* = -0.41, *SE* = 0.15, 95%*CI* [-0.74, -0.15])
  - Indirect effect via comprehensibility (*B* = -0.29, *SE* = 0.13, 95%*CI* [-0.59, -0.07])
- There were no direct (*B* = -0.09, *SE* = 0.50, *p* = .856, 95%*CI* [-1.08, 0.90]) but indirect (*B* = -1.04, *SE* = 0.30, 95%*CI* [-1.65, -0.47]) effects of disrupted plans.
  Examining the mediating effects of the different SOC factors in a parallel mediation analysis showed that all factors were significant mediators:
  - Indirect effect via meaningfulness (*B* = -0.31, *SE* = 0.15, 95%*CI* [-0.65, -0.02])
  - Indirect effect via manageability (*B* = -0.45, *SE* = 0.17, 95%*CI* [-0.82, -0.15])
  - Indirect effect via comprehensibility (*B* = -0.27, *SE* = 0.13, 95%*CI* [-0.57, -0.05])

The pattern of direct and indirect effects did not change when age and gender were included as covariates.
